# Coagulation defects associated with massive blood transfusion: A large multicenter study

**DOI:** 10.3892/mmr.2015.3971

**Published:** 2015-06-22

**Authors:** JIANG-CUN YANG, YANG SUN, CUI-XIANG XU, QIAN-LI DANG, LING LI, YONG-GANG XU, YAO-JUN SONG, HONG YAN

**Affiliations:** 1 Department of Transfusion Medicine, The Third Affiliated Hospital of the Medical College of Xi'an Jiaotong University, Xi'an, Shaanxi 710068, P.R. China; 2 Shaanxi Provincial Center for Clinical Laboratory; Departments of, The Third Affiliated Hospital of the Medical College of Xi'an Jiaotong University, Xi'an, Shaanxi 710068, P.R. China; 3 Dermatology, The Third Affiliated Hospital of the Medical College of Xi'an Jiaotong University, Xi'an, Shaanxi 710068, P.R. China; 4 Laboratory, The Third Affiliated Hospital of the Medical College of Xi'an Jiaotong University, Xi'an, Shaanxi 710068, P.R. China; 5 Urology, The Third Affiliated Hospital of the Medical College of Xi'an Jiaotong University, Xi'an, Shaanxi 710068, P.R. China; 6 Department of Epidemiology and Health Statistics, Medical College of Xi'an Jiaotong University, Xi'an, Shaanxi 710061, P.R. China

**Keywords:** massive transfusion, coagulation, platelet, retrospective analysis, multicenter

## Abstract

The variations in the coagulation indices of patients receiving massive blood transfusion were investigated across 20 large-scale general hospitals in China. The data of 1,601 surgical inpatients receiving massive transfusion were retrospectively collected and the trends in the platelet counts and coagulation indices prior to and at 16 different time points during packed red blood cell (pRBC; after 2–40 units of pRBC) transfusion were evaluated by linear regression analysis. Temporal variations in the means of prothrombin time (PT), international normalized ratio (INR), activated partial thromboplastin time (APTT) and fibrinogen (FIB) concentration were also assessed and the theoretical estimates and actual measurements of the platelet count were compared. The results demonstrated that the platelet count decreased linearly with an increase in the number of pRBC units transfused (Y=150.460−3.041X; R^2^ linear=0.775). Following transfusion of 18 units of pRBC (0.3 units of pRBC transfused per kilogram of body weight), the average platelet count decreased to 71×10^9^/l (<75×10^9^/l). Furthermore, variations in the means of PT, INR, APTT and FIB did not demonstrate any pronounced trends and actual platelet counts were markedly higher than the theoretical estimates. In conclusion, no variations in the means of traditional coagulation indices were identified, however, the platelet count demonstrated a significant linear decrease with an increase in the number of pRBC units transfused. Furthermore, actual platelet counts were higher than theoretical estimates, indicating the requirement for close monitoring of actual platelet counts during massive pRBC transfusion.

## Introduction

Massive blood transfusion is generally defined as the administration of ≥10 units of packed red blood cells (pRBC) to a patient (1,2) or the transfusion of more than one blood volume in 24 h (1,3–5). Acute clinical situations that warrant the administration of massive transfusions include a 50% blood volume loss within 3 h or a blood loss rate of 150 ml/min (3). Massive transfusion is generally necessary in severely injured military personnel or patients with multiple injuries. Such patients often require multiple, complex surgical procedures. A rational blood transfusion protocol can improve the outcome of surgery, whereas unreasonably excessive transfusion can lead to mortality, predominantly due to coagulation disorders, acidosis and hypothermia. The majority of studies published hitherto have been conducted in western countries and on trauma patients (5–10). To the best of our knowledge, no multicenter data are currently available on the effect of massive transfusion on coagulation during the perioperative period in Chinese patients.

In the present study, a retrospective investigation of 1,601 cases of surgical inpatients from 20 large-scale, comprehensive hospitals in different regions of China was undertaken and variations in the coagulation indices of patients were analyzed. The actual measurements of the platelet (PLT) counts in patients receiving massive transfusion against those determined by theoretical calculation were also verified.

## Materials and methods

### Retrospective survey study

#### Study protocol

In the present study, massive blood transfusion was defined as the administration of ≥10 units of pRBC in 24 h. Data was collected from the medical records of surgical inpatients who received massive transfusion at 20 large-scale hospitals in the northwest, southwest, central south, north and northeast regions of China between January 2009 and December 2010. A total of 2,000 copies of the Massive Transfusion Survey Table (hereafter referred to as Survey Table) were distributed to 20 participants in the hospital. Members of the National Massive Transfusion Current Status Investigation Coordination Group (hereafter referred to as the Coordination Group) were responsible for collecting the data from these hospitals using the Survey Table. The data analysis was conducted at Shaanxi Provincial People's Hospital, the Third Affiliated Hospital of the Medical College of Xi'an Jiaotong University (Xi'an, China). The present study was approved by the Ethics Committee of Shaanxi Provincial People's Hospital.

#### Study population

In the present study, the research group included patients who received transfusion of ≥10 units of pRBC over a period of ≤24 h for trauma, cardiac surgery, obstetric conditions or other common surgeries, including orthopedic, thoracic, general, urinary, hepatobiliary and neurological surgery. Patients who received transfusions of <10 U for ≤24 h were assigned to the control group. By contrast, patients with coagulation disorders and/or hepatic failure due to medical causes were excluded from the analysis. Informed consent was obtained from each of the participants.

#### Survey table

The directors of the transfusion departments of the 20 participating hospitals discussed the topic, consulted experts and designed the Survey Table with reference to several international and domestic sources, in accordance with the principle of voluntary participation in this study. A meeting of the Coordination Group was then held, where 35 experts of clinical transfusion, surgery, anesthesia, gynecology and obstetrics, hematology and medical statistics discussed the study protocol and mode of data collection and also perfected and added supplements to the Survey Table. Suitable training was then offered to the investigating staff.

#### Components of the survey table

The survey table comprised the following four sections: i) Clinical and demographic characteristics of the patient, including name, gender, age, body weight, blood type, ethnicity, admission number, admission department, primary diagnosis, secondary diagnosis, pathological diagnosis, nature of surgery and vital signs on admission; ii) details regarding perioperative complications, the clinical condition within 24 h and after 24 h of the transfusion, and the total quantity of blood transfused; iii) the results of the following blood tests performed prior to, within 24 h and after 24 h of transfusion: Routine blood test, coagulation tests, liver function test, kidney function test and arterial blood gas analysis; iv) adverse events due to massive transfusion.

#### Quality control

The Survey Table was initially subjected to a small-scale preliminary test at Shaanxi Provincial People's Hospital so that revisions could be made on the basis of the results and comments of experts to further improve the table. As per the Chinese standards, the protocol for massive transfusion was as follows: One unit of pRBC derived from 200 ml of whole blood with a volume of 140–172 ml; one unit of fresh frozen plasma (FFP) derived from 200 ml of whole blood and a volume of 100 ml; one bag of apheresis PLT of 10 U and a volume of 150–250 ml; and one unit of PLT concentrate derived from 200 ml of whole blood and with a volume of 20–30 ml. One bag of apheresis PLT is 10 units of PLT concentrate. The pRBC were stored at 2–6°C. FFP was stored at ≤−18°C and thawed in a 37°C water bath, for ~10 to 15 min. PLTs were stored at 20–24°C in a platelet shaker.

The main test devices and reagents used were as follows: Sysmex XE-2100/XT-1800i hematology analyzer (Sysmex Corp., Kobe, Japan), Beckman Coulter LH780 Coulter Hematology Analyzer (Beckman Coulter, Inc., Brea, CA, USA); Hitachi 7170A/7180 Biochemical Analyzer (Hitachi, Tokyo, Japan); Roche Modular DP Automatic Biochemical Analyzer (Roche Diagnostics, Indianapolis, IN, USA); Olympus AU640 Biochemical Analyzer (Olympus, Tokyo, Japan); Radiometer ABL-77 Blood Gas Analyzer (Radiometer, Copenhagen, Denmark); Roche Cobas-B123 Blood Gas Analyzer (Roche Diagnostics); Sysmex CA1500/CA7000 Automatic Blood Coagulation Analyzer (Sysmex Corp.). All test reagents used were device-supporting reagents.

Data on the blood tests performed were collected from the laboratory records: Blood routine, coagulation tests, liver function test, kidney function and blood gas analysis. The data were collected for the blood tests performed prior to transfusion and at 16 different time points during the 24-h transfusion (2, 4, 6, 8, 10, 12, 14, 16, 18, 20, 22, 24, 26, 28, 30 and 40 U) and subjected to statistical analysis. The tests were conducted at the laboratory of each participating hospital, which undergoes internal quality control and an external quality assessment conducted by the National Center for Clinical Laboratories (Beijing, China).

#### In vitro test: The effect of the addition of pRBC in vitro following hemodilution on PLT count

Since a large quantity of debris of platelet-like cells was found after performing the PLT count on samples of stored pRBC units, the present study therefore aimed to determine whether the PLT count was affected by the transfusion of pRBC. Thus, the test was performed *in vitro*.

#### Participants

Following approval from the ethics committee and obtaining informed consent, 16 healthy staff members at Shaanxi Provincial People's Hospital, including 10 females and 6 males were included in the present study ranging between 19 and 50 years old. Those with a history of anemia, coagulation disorders, hemorrhage or kidney disease as well as individuals taking anticoagulant drugs within 1 week and females undergoing menstruation were excluded.

#### Preparation of red blood cells

The pRBCs were provided by the Blood Center of Shaanxi Province (Xi'an, China). The blood sample of pRBCs was prepared from the package of the pRBCs stored for 3–5 days.

#### Experimental procedure

Blood (26 ml) from 16 donors was collected in 3.8% sodium citrate. The first 2 ml blood was discarded and the remaining 24 ml blood was used in the experiment. Then, 14 ml was used as a control, following dilution with saline at ratios of 10:0, 9:1, 8:2, 7:3, 6:4, 5:5, 4:6, 3:7, 8:2 and 1:9 (blood versus saline). The other 10 ml was used for the experimental group at dilutions of 7:3, 6:4, 5:5, 4:6, 3:7, 8:2 and 1:9 with saline (blood versus saline). A total of 1 ml of each diluted blood sample was allocated to seven tubes and a different quantity of pRBCs was added to each tube to assess blood routine testing, with reference to the current international guidelines for massive transfusion and surgical blood transfusion (3,5,10,11–15). The critical range of RBC concentration was maintained by ensuring that the hemoglobin level remained at 60–80 g/l. The quantity of pRBCs added was determined according to the results of the experiments regarding the addition of pRBCs under different dilutions (i.e., 100 *μ*l of pRBCs was found to be required for a hemodilution of 30%; 200 *μ*l for 40%; 300 *μ*l for 50%; 400 *μ*l for 60%; 500 *μ*l for 70%; 600 *μ*l for 80% and 700 *μ*l for 90%), which will be published separately.

#### Statistical analysis

Statistical analysis was conducted using SPSS software (version 18.0; SPSS, Inc., Chicago, IL, USA). EpiData (version 3.01; EpiData Association, Odense, Denmark) was used for double data entry verification and database construction. The data on the demographic characteristics and clinical features are expressed as the mean ± standard deviation or as absolute numbers. Categorical variables were analyzed by χ^2^ test, while continuous variables with normal distribution were analyzed by the Shapiro-Wilk test, analysis of variance or the Kruskal-Wallis test, as appropriate. The Bonferroni method was applied for post-hoc tests to determine the significance of the differences between the group that received massive transfusion and the control group that did not. Linear regression was used to describe the association between units of pRBC transfused and PLT count. A two-sided P-value of <0.05 was considered to indicate a statistically significant difference.

## Results

### Patient characteristics

A total of 1,753 of the 2,000 copies of the Survey Table were able to be retrieved from 20 hospitals, at a recovery rate of 87.65%. Following excluding tables with missing information, 1,601 copies (91.33%; 889 male patients; 702 female patients) were used for the analysis. The age of the enrolled patients was 16–91 years (median: 46 years) and weight was 46–105 kg (median: 60 kg). The data regarding age and weight were assessed by the Shapiro-Wilk test (P<0.01) and demonstrated an abnormal distribution; therefore, they were presented as median values. Among the 1,601 patients who received blood transfusion, 1,048 received ≥10 units of pRBC within 24 h (108 died, 940 survived; mortality rate: 10.31%), whereas 553 patients received <10 units of pRBC within 24 h (24 died, 529 survived; mortality rate: 4.34%). The reasons for transfusion in the 1,601 enrolled cases were as follows: Trauma in 268 patients (34 died, 234 survived; mortality rate: 12.69%), cardiac surgery in 383 patients (53 died, 330 survived; mortality rate: 13.84%), general surgery in 876 patients (42 died, 834 survived; mortality rate: 4.79%) and obstetric complications in 74 patients (3 died, 71 survived; mortality rate: 4.05%). The cases of mortality in the present study refer to fatalities occurring during the period of hospitalization. The details of the patient characteristics are provided in Table I.

### PLT variations during massive transfusion

#### PLT count in patients receiving massive blood transfusion

**Figure 1:**
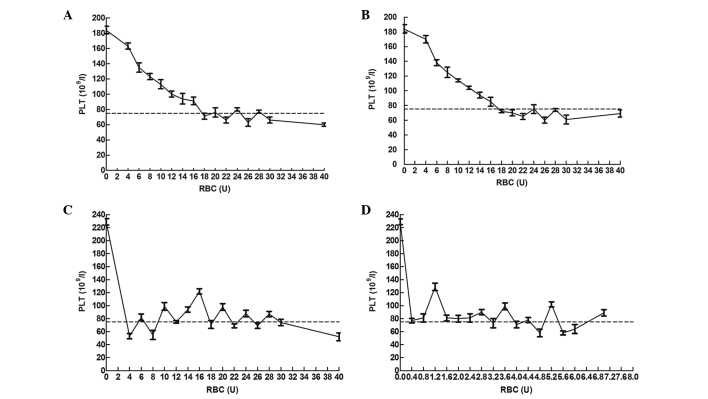
Variations in PLT count during massive blood transfusion. (A) PLT variation for different pRBC units; (B) PLT variation in patients not receiving PLT intervention for different pRBC units; (C) PLT count variations in patients receiving PLT intervention for different pRBC units; (D) RBC units/10 kg weight and PLT count. (---) PLT critical level in patients receiving massive transfusion: PLT count >75×10^9^/l. pRBC, packed red blood cells; PLT, platelet.

Data for the complete blood routine examination at all the different time points considered in the present study were available for 883 of the 1,601 patients enrolled in the present study. Statistical analysis of the data demonstrated that the PLT count in the patients receiving blood transfusion decreased with an increase in the number of pRBC units transfused (Fig. 1A). Following administration of 18 units of pRBC, the PLT count decreased to 71×10^9^/l in patients receiving massive transfusion; Fig. 1B shows the PLT non-intervention group (n=776). When pRBCs reached 18 U, the average PLT counts decreased to 72×10^9^/l; Fig. 1C shows the PLT intervention group (n=107); Fig. 1D shows when patients were transfused with 0.3 units of pRBC per kilogram of body weight (i.e. 3 U/10 kg). The average PLT count decreased to below 75×10^9^/l. With regard to invasive surgery for underweight adults, the critical level of PLTs may be calculated according to the units of pRBC administered and body weight.

#### Linear regression analysis of PLT count measured for different units of pRBC transfused

**Figure 2:**
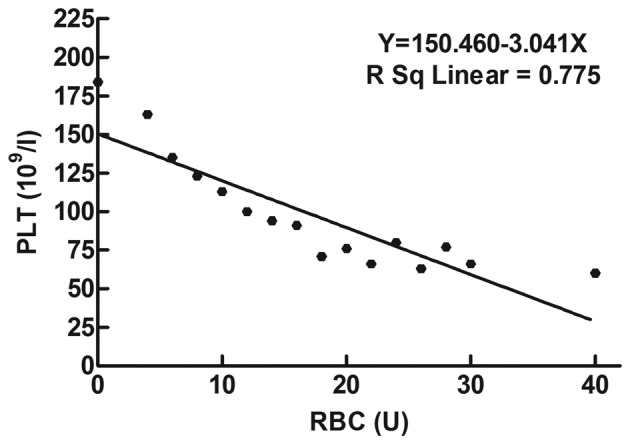
Linear regression trend between units of pRBC transfused and PLT count. pRBC, packed red blood cells; PLT, platelet.

A linear regression analysis of the number of pRBC units transfused and PLT count was performed in 776 patients who received blood transfusion without PLT-intervention, and the results demonstrated that the two parameters were correlated negatively. The linear association was defined as R^2^ linear=0.775, with a regression formula of Y=150.460−3.041X (Fig. 2).

#### PLT count prior to and following the addition of pRBC in the in vitro blood dilution experiment

**Figure 3:**
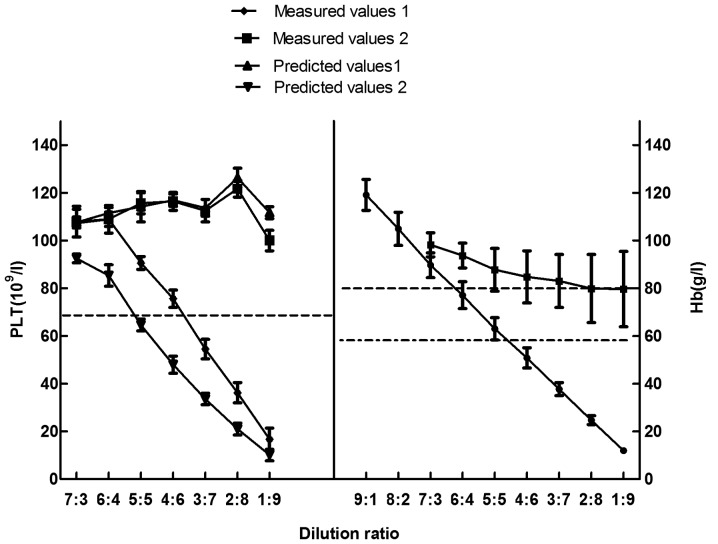
Effect of the number of pRBC units added on platelet count *in vitro*. PLT (measured value 1)=following hemodilution. PLT (measured value 2)=value following addition of pRBC suspension following hemodilution. PLT (predicted value 1)=platelet count without addition of pRBC. PLT (predicted value 2)=platelet count following addition of pRBC. PLT (predicted value 1)=PLT (0) × dilution × 1,000/(1,000+M). PLT (predicted value 2)=PLT (0) × dilution × 1,000/(1,000+M) + PLT (pRBC) × M/(1,000+M). 16U pRBC blood routine indices: RBC=(6.29±1.05) ×10^12^/l; Hct=0.5898±0.1 l/l; Hb=190.4±39.01 g/l; PLT=(239.8−135.29) ×10^9^/l. (---) PLT critical level in patients receiving massive transfusion: PLT count >75×10^9^/l. (---/---) Hb critical level: 60–80 g/l. pRBC, packed red blood cells; PLT, platelet; Hb, hemoglobin; Hct, hematocrit.

The data of 16 unrelated healthy volunteers were analyzed; pRBC were added at different dilutions to maintain the range of hemoglobin concentration at 60–80 g/l. The results demonstrated that the PLT count decreased with an increase in the hemodilution. It was found that administration of pRBC for the correction of anemia also corrected the PLT count. Further analysis revealed that the results of the automated device for counting blood cells were affected by the addition of pRBC (Fig. 3). RBC=(6.29±1.05) ×10^12^/l, hematocrit=0.5898±0.1 l/l, hemoglobin=190.4±39.01 g/l and PLT count=(239.8±135.29) ×10^9^/l. These results suggested that RBC in the pRBC contained PLT-like cell fragments.

### Variation in coagulation parameters of patients with massive transfusion

#### Supplementation of FFP (coagulation factor supplement)

**Figure 4:**
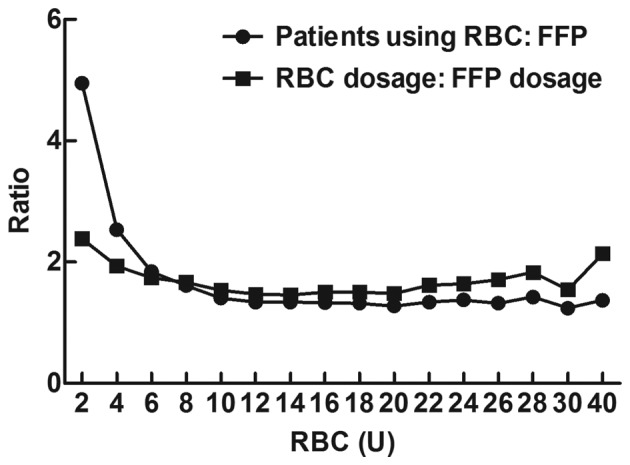
Ratio of the RBC dosage and FFP dosage for different units of pRBC transfused. pRBC, packed red blood cells; FFP, fresh frozen plasma.

The data of 1,601 cases enrolled in the present study demonstrated that all hospitals administered FFP to supplement pRBC transfusion. When RBC transfusion was 2–8 U, the ratio of patients transfused with RBC and plasma was 4.88:1–1.61:1 (1601:328–1313:816), and at RBC transfusions of 10–30 U, the ratio of patients transfused with pRBC and plasma was 1.38:1–1.24:1 (1048:759–62:50). This implies that for transfusion of 10–30 units of pRBC, the ratio of patients transfused with plasma accounted for 1:1.38–1:1.24 (759:1048–50:62) of those transfused with pRBC, while the quantity of plasma transfused accounted for 1:1.54–1:1.55 (6939.5:10700–1159.5:1800) that of pRBC transfused (Fig. 4).

#### Variation in coagulation indices in patients receiving massive blood transfusion

**Figure 5:**
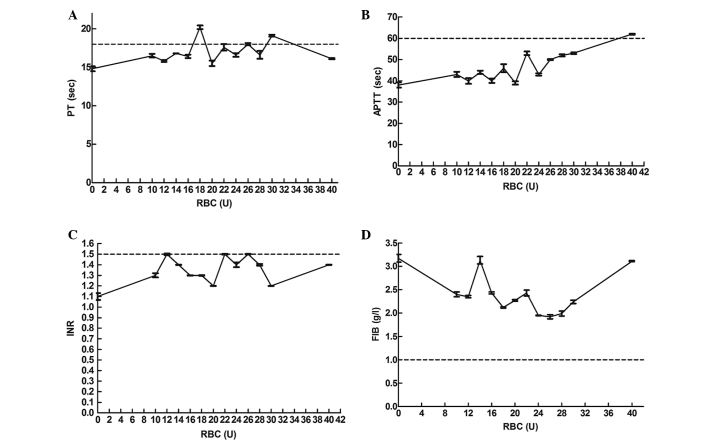
Variation in coagulation indices in patients receiving massive transfusion. (A) pRBC units transfused and PT; (B) pRBC units transfused and APTT; (C) pRBC units transfused and INR; (D) pRBC units transfused and FIB. (---) Abnormal coagulation is indicated by a PT >18 sec, APTT >60 sec, INR >1.5 and FIB <1.0 g/l. pRBC, packed red blood cells; INR, international normalized ratio; PT, prothrombin time; APTT, activated partial thromboplastin time; FIB, fibrinogen concentration.

The results of the present study indicated that the prothrombin time (PT) varied between 15 and 20 sec when RBC transfusion was 2–40 U (Fig. 5A). Activated partial thromboplastin time (APTT) demonstrated a gradual extension with an increase in the number of RBC units transfused, reaching 60 sec when 40 units were transfused (Fig. 5B). For pRBC transfusions of 2–40 U, the international normalized ratio (INR) and fibrinogen (FIB) concentration varied within the range of 1.0–1.5 (Fig. 5C) and 2–3 g/l (Fig. 5D), respectively.

## Discussion

Transfusion is important for treating patients presenting with emergent, potentially fatal conditions. The timely administration of blood transfusion in sufficient quantities is critical to prevent mortality in patients with severe blood loss. However, the mortality rate in trauma patients receiving massive transfusion is high, ranging between 19 and 70% (16–18). In the present study comprising 1,048 cases of massive blood transfusion (administration of ≥10 units of pRBC within 24 h), the mortality rate was 10.31%, which is lower than that reported previously (16–18). This discrepancy may be attributed to a few characteristic features of the present study. The 20 participating medical institutions in the present study are large-scale general hospitals, which are well-equipped for life-saving procedures. Among the cases enrolled, only a few were of trauma; the majority included patients who had undergone general surgery with good preoperative preparation of the patient. The transfusion protocol adopted was immediate administration of FFP at a high concentration along with pRBC transfusion to correct the coagulation status at the initial stage. Further studies are necessary to determine whether the administration of FFP may have contributed to the reduced mortality rate in the present study. According to the guidelines for massive transfusion or surgical blood transfusion (3,5,10,11–15), the PLT count should be maintained >75×10^9^/l. The present study demonstrated that the infusion of pRBC has a marked effect on the PLT count in cases of massive transfusion. On transfusion of 18, 25 and 0.3 units per kilogram of body weight (0.3 U/kg) of pRBC, the average PLT count decreased to 71, 60 and <75×10^9^/l, respectively. In addition, in the present study 107 patients received PLT therapy with pRBC transfusion. Thrombocytopenia occurs in patients during the initial stage of RBC transfusion (thrombocytopenia caused by disease or prior to transfusion). PLT intervention therapy in the initial transfusion stage and maintaining the PLT count between 53×10^9^/l–122×10^9^/l ensures smooth operation of invasive surgery.

A study by Miller *et al* demonstrated that a reduction in PLT count due to dilution is the cause of coagulation disorders during massive transfusion and that the actual measured value of PLT is higher than the theoretically calculated value, following blood dilution (19). The actual measured PLT count was 60×10^9^/l after 25 units of pRBC was transfused, however, the calculated value was ~20×10^9^/l. A linear regression analysis was conducted on the actual PLT count measured following transfusion of different units of pRBC and found that the decrease in the PLT count correlated with the increase in the number of pRBC units transfused in a linear fashion (Y=150.460−3.041X, R^2^ linear=0.775). The decrease in the PLT counts noted in the present study was lower than that reported by Counts *et al* (<100×10^9^/l) following transfusion of 18 units of pRBC (20). The difference between the actual PLT count and the value predicted by theoretical calculation may be attributed to the release of PLTs stored in the internal organs, including the spleen, lung and liver or early release of PLTs into the blood circulation by the mobilization of the marrow, thereby offsetting blood dilution (21). It was hypothesized that this increase in the actual count measured may be due to the release of PLT fragments or PLT-like substances into the circulation of the patient via the transfused pRBC units. This explanation is based on the results of our *in vitro* experiments to determine whether the addition of pRBC affected the PLT count.

Following the addition of pRBC for the correction of anemia, the PLT counts increased. However, the PLT counts are theoretically expected to decrease with an increase in the hemodilution. It was hypothesized that the large number of RBC transfused may contain PLT-like cellular fragments. Therefore, it was recommended that close attention should be paid by clinicians to the PLT levels in patients receiving massive transfusion and that the actual measurement of the PLT count be relied upon rather than theoretical calculation.

Previous studies have indicated that immediate administration of FFP at high concentrations or transfusion with an appropriate ratio of plasma to RBC (FFP: RBC=1:1−2) can reduce the mortality rate of patients receiving massive blood transfusion (6–8,17,22–24). The abovementioned ratio was maintained in the present study, implying that for transfusion of 10–30 units of pRBC, the ratio of patients transfused with plasma accounted for 1:1.38–1:1.24 and the quantity of FFP transfused accounted for 1:1.54–1:1.55 of RBC transfused.

Previous studies on massive transfusion (25) have used the criteria of PT >18 sec and APTT >60 sec or INR >1.5 and FIB <1.0 g/l to define abnormal coagulation. Coagulation is considered to be normal if FIB is >0.8–1.0 g/l and PT or APTT is <1.5-times the normal (3,5,10,11–15). No alterations in the mean values of PT, APTT, INR and FIB were found during massive blood transfusion. This may be associated with the infusion of a high concentration of FFP along with pRBC transfusion in the present study. Several studies have indicated that when coagulation factors are reduced to 20–30% of the normal level, the patient can endure invasive surgery and a PT >30–40% suggests that the levels of coagulation factors are safe (5,10). If APTT is >1.8-times the normal value or INR>1.5–1.8-times the normal value, coagulation disorders are highly likely (20,26). The results demonstrated that in the group with a FFP:RBC ratio of 1:1.54, the differences between the mean value of the coagulation indices at different time points was not clear. It is possible that the administration of FFP to supplement pRBC transfusion may have prevented coagulation disorders. The results of the present study suggest that supplementation of FFP (coagulation factor) and PLT during massive transfusion may ensure safety during surgery. In addition, studies suggest that thromboelastography may be more useful than the traditional indices of PT/INR and APTT/FIB to evaluate the coagulation status of patients receiving massive transfusion (1–3,5,11,13).

Of note, the present study has certain limitations. For example, this was a retrospective study. Subsequent studies with prospective design are required to overcome this limitation. Furthermore, the transfusion protocol of immediate administration of FFP with pRBC requires further validation.

In conclusion, the present study revealed that in patients undergoing massive blood transfusion, the PLT count declined with an increase in the number of pRBC units transfused, with a linear association between the two parameters (Y=150.460−3.041X, R^2^ linear=0.775). The present study also demonstrated that the actual PLT count was significantly greater than that estimated theoretically in patients undergoing massive blood transfusion; therefore, the latter estimation should only be used as a supplementary reference. Variations in the mean values of the traditional coagulation indices (PT, APTT, INR and FIB) assessed in the present study were not apparent, which may be due to the transfusion of FFP (supplement coagulation factor) along with pRBC. *In vitro* experiments also revealed that the addition of pRBC affected the PLT count.

## Figures and Tables

**Table I:** Baseline data of 1,601 patients receiving massive transfusion.

Demographics and clinical data	<10 units of pRBC	≥10 units of pRBC	P-value
Demographics			
Number of patients, n (%)	553 (34.5)	1,048 (65.5)	
Age, years (mean ± SD)	46.5±18.2	44.9±16.7	
Males, n (%)	300 (300/553)	402 (402/1048)	
Weight, kg (mean ± SD)	56.6±13.9	58.5±11.4	
Patients suffering from trauma, n (%)	81 (30.2)	187 (69.8)	
Patients who underwent cardiac surgery, n (%)	116 (30.3)	267 (69.7)	
Patients who underwent general surgery, n (%)	335 (38.2)	541 (61.8)	
Patients with obstetric complications, n (%)	21 (28.4)	53 (71.6)	
Clinical data (prior to transfusion)			
Respiration, n/min (mean ± SD)	20.3±3.5	20.5±3.6	0.043^a^
Pulse, n/min (mean ± SD)	94.1±69.8	92.5±54.3	0.452^a^
SBP, mmHg (mean ± SD)	113.5±24.7	112.8±30.2	0.020^a^
Temperature, °C (mean ± SD)	36.6±1.0	36.5±0.7	0.319^a^
RBC, ×10^12^/l (mean±SD)	3.8±1.0	3.8±1.1	0.323^a^
Hb, g/l (mean ± SD)	114.3±30.2	117.4±43.2	0.213^a^
Hct as % (mean ± SD)	21.2±17.7	16.6±17.6	0.834^a^
PLT, ×10^9^/l (mean±SD)	179.5±91.5	175.6±98.9	0.324^a^
PT, sec (mean ± SD)	13.7±6.0	14.1±5.8	0.173^a^
APTT, sec (mean ± SD)	33.6±11.7	36.3±24.2	0.006^a^
TT, sec (mean ± SD)	17.1±12.8	17.5±7.1	0.529^a^
INR (mean ± SD)	1.3±2.1	1.2±1.1	0.041^a^
FIB, g/l (mean ± SD)	11.3±44.4	11.0±46.6	0.801^a^
Clinical data (following transfusion)			
Length of hospital stay, days (mean ± SD)	24.9±14.3	29.8±23.9	0.000^a^
Length of stay in ICU, days (mean ± SD)	3.8±3.5	8.7±23.4	0.006^a^
Surgery time, h (mean ± SD)	2.5±3.2	3.7±3.9	0.000^a^
pRBC in 24 h, units (median)	9	25	0.000^b^
FFP in 24 h, units (median)	8	20	0.000^b^
PLT in 24 h, units (median)	10	6	0.009^b^
pRBC in 72 h, units (median)	20	18	0.202^b^
FFP in 72 h, units (median)	14	13	0.499^b^
PLT in 72 h, units (median)	8	8	0.873^b^

APTT, activated partial thromboplastin time; FIB, fibrinogen concentration; Hb, hemoglobin concentration; ICU, intensive care unit; INR, international normalized ratio; PLT, platelet count; PT, prothrombin time; RBC, red blood cell count; SBP, systolic blood pressure; TT, thrombin time; SD, standard deviation; Hb, hemoglobin; Hct, hematocrit; pRBC, packed red blood cells; FFP, fresh frozen plasma.

a Analysis of variance;

b Kruskal-Wallis test.
